# 35 The Impact of Hand Burns on Strength; An Analysis of the ACT Database

**DOI:** 10.1093/jbcr/irae036.035

**Published:** 2024-04-17

**Authors:** Renée Warthman, Curt C Bay, Whitney Pirsig, Derek Murray, Karen J Richey, Kevin N Foster

**Affiliations:** Arizona Burn Center at Valleywise Health, Phoenix, AZ; A.T. Still University, Mesa, AZ; Valleywise Health Medical Center - AZ Burn Center, Gilbert, AZ; Valleywise, Phoenix, AZ; Arizona Burn Center Valleywise Health, Phoenix, AZ; Arizona Burn Center at Valleywise Health, Phoenix, AZ; A.T. Still University, Mesa, AZ; Valleywise Health Medical Center - AZ Burn Center, Gilbert, AZ; Valleywise, Phoenix, AZ; Arizona Burn Center Valleywise Health, Phoenix, AZ; Arizona Burn Center at Valleywise Health, Phoenix, AZ; A.T. Still University, Mesa, AZ; Valleywise Health Medical Center - AZ Burn Center, Gilbert, AZ; Valleywise, Phoenix, AZ; Arizona Burn Center Valleywise Health, Phoenix, AZ; Arizona Burn Center at Valleywise Health, Phoenix, AZ; A.T. Still University, Mesa, AZ; Valleywise Health Medical Center - AZ Burn Center, Gilbert, AZ; Valleywise, Phoenix, AZ; Arizona Burn Center Valleywise Health, Phoenix, AZ; Arizona Burn Center at Valleywise Health, Phoenix, AZ; A.T. Still University, Mesa, AZ; Valleywise Health Medical Center - AZ Burn Center, Gilbert, AZ; Valleywise, Phoenix, AZ; Arizona Burn Center Valleywise Health, Phoenix, AZ; Arizona Burn Center at Valleywise Health, Phoenix, AZ; A.T. Still University, Mesa, AZ; Valleywise Health Medical Center - AZ Burn Center, Gilbert, AZ; Valleywise, Phoenix, AZ; Arizona Burn Center Valleywise Health, Phoenix, AZ

## Abstract

**Introduction:**

Although hand burns comprise a small TBSA percentage, they can have significant functional implications. Multiple studies show grip strength as a predictor of health and quality of life. A decrease in grip strength has been reported in multiple populations with hospital admission; however, there is limited evidence concerning how burns affect hand strength. The purpose of this study was to examine the impact of burns on grip strength, measured at hospital discharge.

**Methods:**

This study is a retrospective review of data from the prospective Burn Patient Acuity Demographics, Scar Contracture and Rehabilitation Treatment Related to Patient Outcome Study (ACT). Patients had grip strength measured of both hands 3 times consecutively at discharge. Data related to grip strength of patients with burn injuries were compared to age- and sex-matched normative values. Burn characteristics, including presence of hand burns, skin grafting, and length of stay were evaluated.

**Results:**

There were 307 participants in ACT; 195 (63.5%) had hand burns and met inclusion for analyses. Among those with hand burns, the majority were male (72.8%), right-hand dominant (84.1%), mean ±SD age was 42.9±17.0; LOS, 23.3±22.8 days; and TBSA, 14.5%±13.3. Among patients with hand burns, 112 (57.4%) received skin grafts to at least one hand. Expressed as the percentage of grip strength loss relative to normative data, those with a burn injury to the hand averaged -55.8±33.4%, while those with no burns to the hand averaged -25.7%±36.3%, p < .001. Patients with a skin graft to the hand averaged -65.8±27.2% grip strength, while those with hand burns, but no grafts averaged -36.1%±35.5%, p < .001. Decreased grip strength is associated with increased length of stay, r= .21, p < .001.

**Conclusions:**

This study of hand strength in burn survivors at the time of hospital discharge corroborates previous reports regarding nosocomial strength loss but specific to the hand. Overall, patients hospitalized with burn injuries lose hand grip strength but more-so if a hand is burned and furthermore if a hand is skin grafted.

**Applicability of Research to Practice:**

Understanding of the impact of burns on grip strength may help guide clinical decision making, allowing therapists treating those with burn injuries to anticipate deficits and subsequent functional impact. Expectation of a loss of grip strength in patients without burns to the hands should inform treatment planning during acute hospitalization to promote improved function and quality of life.

